# Functional and structural characterization of allosteric activation of phospholipase Cε by Rap1A

**DOI:** 10.1074/jbc.RA120.015685

**Published:** 2021-01-13

**Authors:** Monita Sieng, Arielle F. Selvia, Elisabeth E. Garland-Kuntz, Jesse B. Hopkins, Isaac J. Fisher, Andrea T. Marti, Angeline M. Lyon

**Affiliations:** 1 Department of Chemistry, Purdue University, West Lafayette, Indiana, USA; 2 Biophysics Collaborative Access Team, Illinois Institute of Technology, Advanced Photon Source, Argonne National Laboratory, Lemont, Illinois, USA; 3 Department of Biological Sciences, Purdue University, West Lafayette, Indiana, USA

**Keywords:** calcium intracellular release, cardiovascular disease, cell signaling, G protein, phosphatidylinositol signaling, phospholipase C, Ras-related protein 1 (Rap1), diacylglycerol, protein kinase C (PKC), small-angle X-ray scattering (SAXS), conformational change, structural biology, membrane enzyme

## Abstract

Phospholipase Cε (PLCε) is activated downstream of G protein–coupled receptors and receptor tyrosine kinases through direct interactions with small GTPases, including Rap1A and Ras. Although Ras has been reported to allosterically activate the lipase, it is not known whether Rap1A has the same ability or what its molecular mechanism might be. Rap1A activates PLCε in response to the stimulation of β-adrenergic receptors, translocating the complex to the perinuclear membrane. Because the C-terminal Ras association (RA2) domain of PLCε was proposed to the primary binding site for Rap1A, we first confirmed using purified proteins that the RA2 domain is indeed essential for activation by Rap1A. However, we also showed that the PLCε pleckstrin homology (PH) domain and first two EF hands (EF1/2) are required for Rap1A activation and identified hydrophobic residues on the surface of the RA2 domain that are also necessary. Small-angle X-ray scattering showed that Rap1A binding induces and stabilizes discrete conformational states in PLCε variants that can be activated by the GTPase. These data, together with the recent structure of a catalytically active fragment of PLCε, provide the first evidence that Rap1A, and by extension Ras, allosterically activate the lipase by promoting and stabilizing interactions between the RA2 domain and the PLCε core.

Phospholipase C (PLC) enzymes hydrolyze phosphatidylinositol lipids from cellular membranes in response to diverse cellular signals (1, 2). These proteins cleave phosphatidylinositol 4,5-bisphosphate (PIP_2_) at the plasma membrane, producing the second messengers inositol 1,4,5-triphosphate (IP_3_) and diacylglycerol, which increase Ca^2+^ in the cytoplasm and activate PKC, respectively. However, some PLC subfamilies, such as PLCε, also hydrolyze other phosphatidylinositol phosphate species at internal membranes (3, 4, 5, 6). Thus, PLC enzymes regulate multiple pathways from different subcellular locations (1, 2).

PLCε is required for maximum Ca^2+^-induced Ca^2+^ release in the cardiovascular system (7, 8). In pathological conditions such as heart failure, PLCε expression and activity are increased, promoting overexpression of genes involved in cardiac hypertrophy through a PKC-dependent mechanism (4, 5, 6, 9, 10). Like other PLCs, PLCε contains conserved core domains, including a PH domain, four EF hand repeats (EF1–4), a catalytic TIM barrel domain, and a C2 domain (1). However, unique N- and C-terminal regulatory regions flank this core. The N-terminal region contains a CDC25 domain that is a guanine-exchange factor for the Rap1A GTPase (11, 12, 13), whereas the C-terminal region contains two Ras association (RA) domains, RA1 and RA2 (Fig. 1*A*). Recent functional analysis of a catalytically active PLCε fragment containing the EF3-RA1 domains confirmed that the CDC25, PH, and RA2 domains, as well as EF1/2, are dispensable for expression and activity. The structure of this fragment revealed that the RA1 domain and the linker connecting the C2 and RA1 domains (C2-RA1) form extensive interactions with EF3/4, the TIM barrel, and the C2 domain. Thus, in PLCε, the core is expanded to encompass the PH-RA1 region.

**Figure 1:**
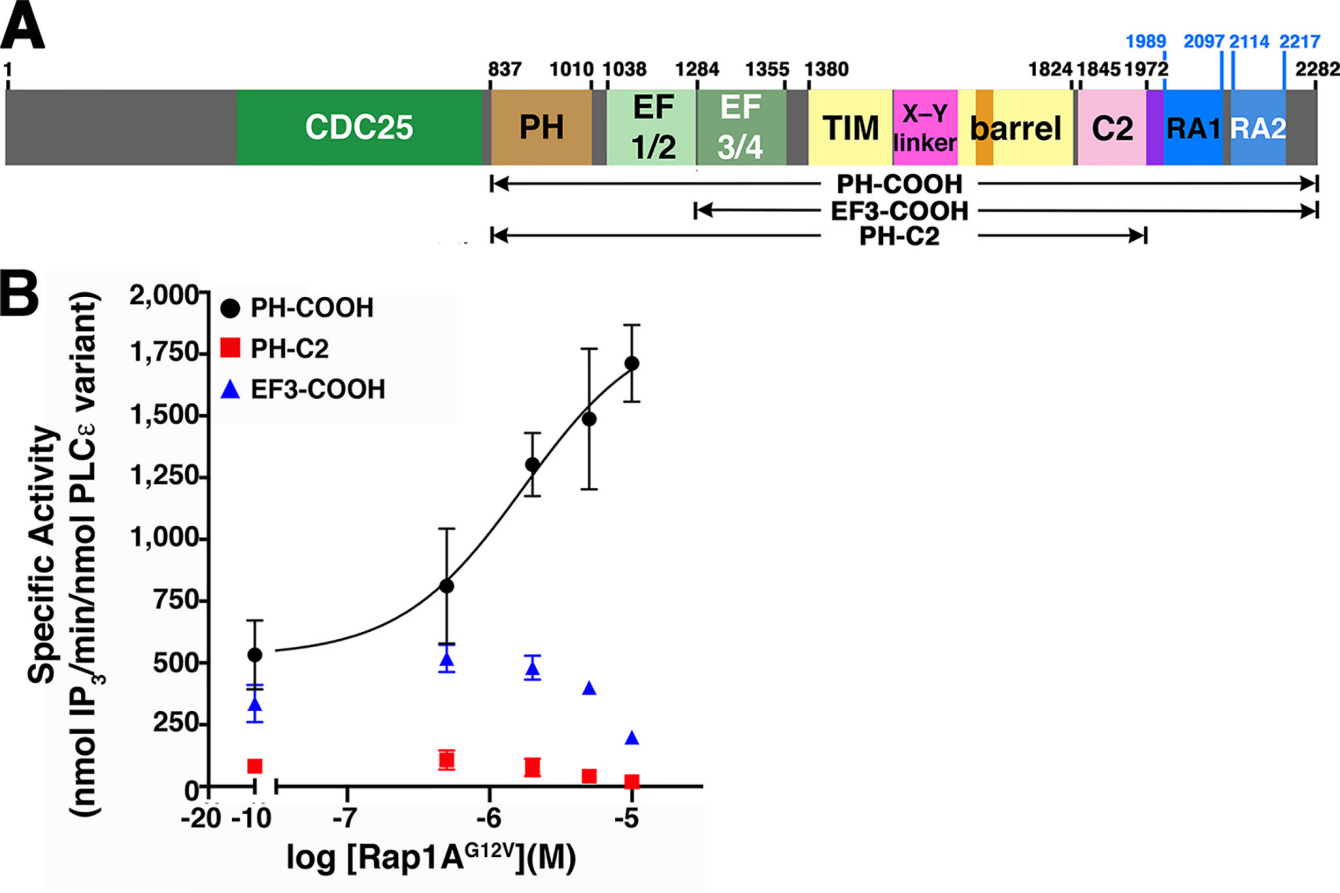
**Multiple domains in PLCε are required for Rap1A^G12V^-dependent activation.***A*, domain diagram of rat PLCε. The Y-box insertion and C2-RA1 linker are shown in *orange* and *purple*, respectively. *Numbers* above the diagram correspond to the domain boundaries most relevant to this work, with the variants under study shown below. *B*, PLCε PH-COOH (*black circles*) is activated by Rap1A^G12V^ in a concentration-dependent manner. In contrast, PH-C2 (*red squares*) and EF3-COOH (*blue triangles*) are not activated at any concentration of Rap1A^G12V^ tested. The data represents at least two independent experiments performed in duplicate. *Error bars* represent S.D.

The PLCε RA1 and RA2 domains also contribute to subcellular localization and activation via direct interactions with the scaffolding protein mAKAP (muscle-specific protein kinase A–anchoring protein) and the Rap1A and Ras GTPases, respectively (1, 6, 16, 17, 18). Of these, the activation of PLCε by Rap1A has been most studied. Stimulation of β-adrenergic receptors in the cardiovascular system activates adenylyl cyclase, increasing cAMP, which in turn activates Epac (exchange protein activated by cAMP). Epac catalyzes nucleotide exchange on Rap1A, which binds the RA2 domain, thereby recruiting and allosterically activating PLCε at the Golgi and perinuclear membranes for phosphatidylinositol 4-phosphate hydrolysis (6, 7, 8, 9, 10). The guanine-exchange factor activity of PLCε for Rap1A results in the formation of a local pool of activated Rap1A, establishing a feed-forward activation loop (19, 20, 21). Sustained signaling through this mechanism is thought to be one of the key processes underlying pathologic cardiac hypertrophy (3, 4, 5, 9, 10).

Ras-dependent activation of PLCε has been partially characterized, and its mechanism requires both membrane association and allosteric components (14, 17). Although Rap1A is anticipated to share a similar mechanism of activation, it has not yet been shown to allosterically activate PLCε, and the mechanism by which this would occur is not known. We hypothesized that Rap1A binding works in concert with the membrane surface to promote interdomain contacts in PLCε that stabilize a more catalytically competent state, as has been reported for Gα_q_-dependent activation of the related PLCβ enzyme (22). We found that multiple domains of PLCε, in addition to RA2, are required for activation by constitutively active Rap1A. We also identified hydrophobic residues on the surface of the RA2 domain, distant from the GTPase-binding site, that are also essential for Rap1A activation. Finally, we used small-angle X-ray scattering (SAXS) to show that Rap1A binding induces and stabilizes long-range structural changes in PLCε that are consistent with the 3D architecture of the enzyme. Together, these results provide new insights into the structure and molecular mechanism of allosteric activation of PLCε by Rap1A.

## Results

### Rap1A-dependent activation of PLCε requires multiple domains of the lipase

Rap1A-dependent activation of PLCε has been demonstrated in cell-based assays, but not using purified components (20, 21, 23). Because full-length PLCε has not been purified in sufficient quantities for biochemical analysis, we relied on the PLCε PH-COOH variant for these studies (Fig. 1*A*) (24), which retains both RA domains and is thus expected to be responsive to Rap1A. In a liposome-based activity assay, the addition of constitutively active and prenylated Rap1A^G12V^ increased the specific activity of PH-COOH ∼3-fold over basal, with a maximum specific activity of 1,900 ± 300 nmol IP_3_/min/nmol PLCε variant (Fig. 1*B*, Table 1, and Table S1), which is similar to the fold activation reported in cell-based assays using full-length PLCε (17, 23, 25).

**Table 1:** Stability, basal activity, and fold-activation by Rap1A of PLCε variants The data represent at least two independent experiments performed in duplicate ± S.D. The results are based on one-way analysis of variance followed by Dunnett's multiple comparisons test *versus* PLCε PH-COOH.

PLCε variant	*T*_m_	Basal specific activity^a^	Fold activation by Rap1A^G12V^ (*n*)
	° *C (n)*	*nmol IP_3_/min/nmol PLC*ε *variant) (n)*	
PH-COOH^b^	51.3 ± 0.72 (6)	360 ± 120 (9)	3.0 (4)
EF3-COOH	51.7 ± 0.12 (4)	450 ± 50 (3)	N/A (3)
PH-C2^b^	48.3 ± 1.00 (4)^c^	80 ± 20 (5)^c^	N/A (2)
PH-COOH K2150A	50.5 ± 0.86 (3)	160 ± 30 (4)^d^	1.8 (2)
PH-COOH K2152A	49.6 ± 0.79 (3)	240 ± 20 (3)	0.9 (3)
PH-COOH Y2155A	49.8 ± 1.06 (3)	250 ± 40 (5)	1.4 (3)
PH-COOH L2158A	51.2 ± 0.39 (3)	270 ± 100 (3)	1.0 (3)
PH-COOH L2192A	51.5 ± 0.71 (3)	270 ± 50 (3)	1.3 (3)
PH-COOH F2198A	51.5 ± 0.45 (3)	280 ± 100 (3)	1.2 (3)


a Maximum specific activities were measured at a single time point.

b *T*_m_ and specific activity for these variants were previously reported and are included here for comparison (24).

c *p* ≤ 0.0002.

d *p* ≤ 0.0028.

The RA2 domain is expected to be the primary binding site for Rap1A^G12V^, but other regions of PLCε may also be required for activation. Given that PLCε domains EF3-C2 are essential for basal activity (26, 27, 28, 29), we focused on the roles of the N-terminal PH domain and EF1/2 in Rap1A-dependent activation. The PLCε EF3-COOH fragment lacks these elements and has similar stability and basal activity to PH-COOH (Table 1 and Fig. S1). As a negative control, we also investigated the PH-C2 variant, which has reduced stability and activity relative to PH-COOH (Table 1 and Fig. S1) (24) and lacks both RA domains. Indeed, PLCε PH-C2 was not activated by Rap1A^G12V^ at any concentration tested, consistent with the absence of the RA2 domain (Fig. 1*B* and Table 1). Surprisingly, PLCε EF3-COOH also showed no activation, indicating that the PLCε PH domain and/or EF1/2 are necessary for Rap1A-dependent activation (Fig. 1*B*, Table 1, and Table S1).

### Hydrophobic residues on the RA2 domain surface are required for Rap1A-dependent activation

Because our data were consistent with multiple PLCε domains contributing to Rap1A^G12V^-dependent activation, we hypothesized that Rap1A allosterically activates PLCε by promoting or stabilizing potentially long range intra- and interdomain interactions within the lipase. Because both Ras and Rap1A bind to and activate the enzyme via the RA2 domain, the surface of the RA2 domain is the most likely candidate to mediate these interactions. To date, only two residues on the RA2 domain have been characterized with respect to activation by GTPases. Mutation of Lys^2150^ and/or Lys^2152^ (*Rattus norvegicus* numbering) decreases basal activity and eliminates G protein–dependent activation in cell-based studies (17, 23). Based on the structure of activated H-Ras bound to the isolated RA2 domain, Lys^2150^ makes an electrostatic interaction with switch II in the GTPase, whereas Lys^2152^ positively contributes to the local electrostatic environment (14).

**Figure 2:**
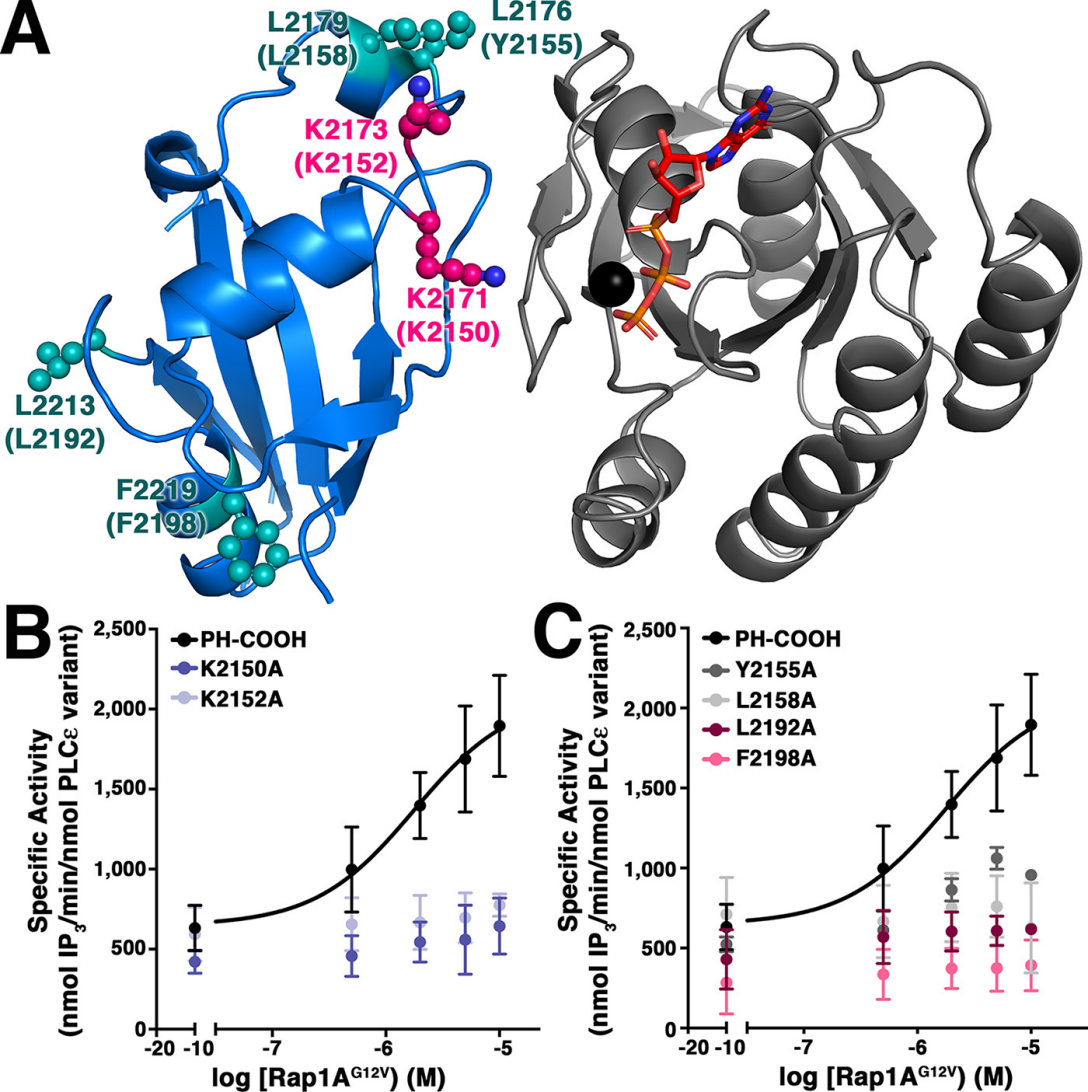
**Hydrophobic residues on the surface of the RA2 domain are critical for activation.***A*, the structure of H-Ras (*gray*) bound to the RA2 domain (*blue*, PDB entry 2C5L (14)) reveals conserved, hydrophobic residues (*teal spheres*) involved in crystal lattice contacts. Lys^2171^ and Lys^2173^ (*hot pink spheres*) were previously reported to be required for Rap1A-dependent activation. *R. norvegicus* residues are in *parentheses*. GTP is shown as *orange sticks*, and Mg^2+^ is shown as a *black sphere*. *B* and *C*, mutation of the Lys^2150^ or Lys^2152^ to alanine eliminates activation by Rap1A^G12V^*in vitro* (*B*), as does mutation of the conserved hydrophobic residues distant from the Rap1A binding surface (*C*).

We hypothesized that residues involved in allosteric activation on the RA2 domain would be surface-exposed and conserved, and most likely hydrophobic residues. Using the H-Ras–RA2 structure to model the Rap1A–RA2 interaction (Fig. 2*A*; PDB entry 2C5L (14)), we identified four conserved hydrophobic residues on the surface of the RA2 domain involved in lattice contacts in the crystal structure that did not interact with the GTPase. These residues, Tyr^2155^, Leu^2158^, Leu^2192^, and Phe^2198^ (*R. norvegicus* numbering) were individually mutated to alanine in the background of the PLCε PH-COOH variant, and their melting temperatures (*T*_m_) and basal activities were determined. As controls, we also expressed, purified, and characterized the PH-COOH Lys^2150^ and Lys^2152^ point mutants, because they should be insensitive to Rap1A-dependent activation (17, 23).

The PH-COOH Y2155A, L2158A, L2192A, and F2198A mutants all had *T*_m_ values comparable with that of PH-COOH (15, 30), and basal specific activities within ∼2-fold of PH-COOH (Table 1 and Fig. S1), demonstrating that they are properly folded. K2150A and K2152A also had *T*_m_ values comparable with that of PH-COOH, but K2150A had ∼2-fold lower basal activity, consistent with previous reports (Table 1 and Fig. S1) (17, 23). We then tested the ability of Rap1A^G12V^ to activate the point mutants in a liposome-based activity assay. The K2150A and K2152A mutants were insensitive to activation by Rap1A^G12V^, consistent with their proposed role in binding GTPases (Fig. 2*B* and Table 1) (17, 23). Mutation of the hydrophobic surface residues also decreased or eliminated Rap1A^G12V^-dependent activation at all concentrations tested (Fig. 2*C*, Table 1, and Table S1). Thus, these hydrophobic residues appear to play a critical role in this mechanism, independent of GTPase binding.

### Rap1A^G12V^ binding to PLCε induces and stabilizes unique conformational states

Our domain deletion and site-directed mutagenesis analyses identified roles for the PH domain, EF1/2, and hydrophobic residues on the RA2 surface in Rap1A^G12V^-dependent activation. To gain structural insight into how these elements that are distant in primary structure contribute to activation by Rap1A^G12V^, we used SAXS to compare the solution structures of the PLCε PH-COOH and EF3-COOH variants alone and in complex with Rap1A^G12V^. These variants were chosen because they formed stable complexes with Rap1A^G12V^ that could be isolated by size-exclusion chromatography (SEC), but only PH-COOH has increased lipase activity upon binding of the GTPase (Fig. 1 and Table 1).

**Figure 3:**
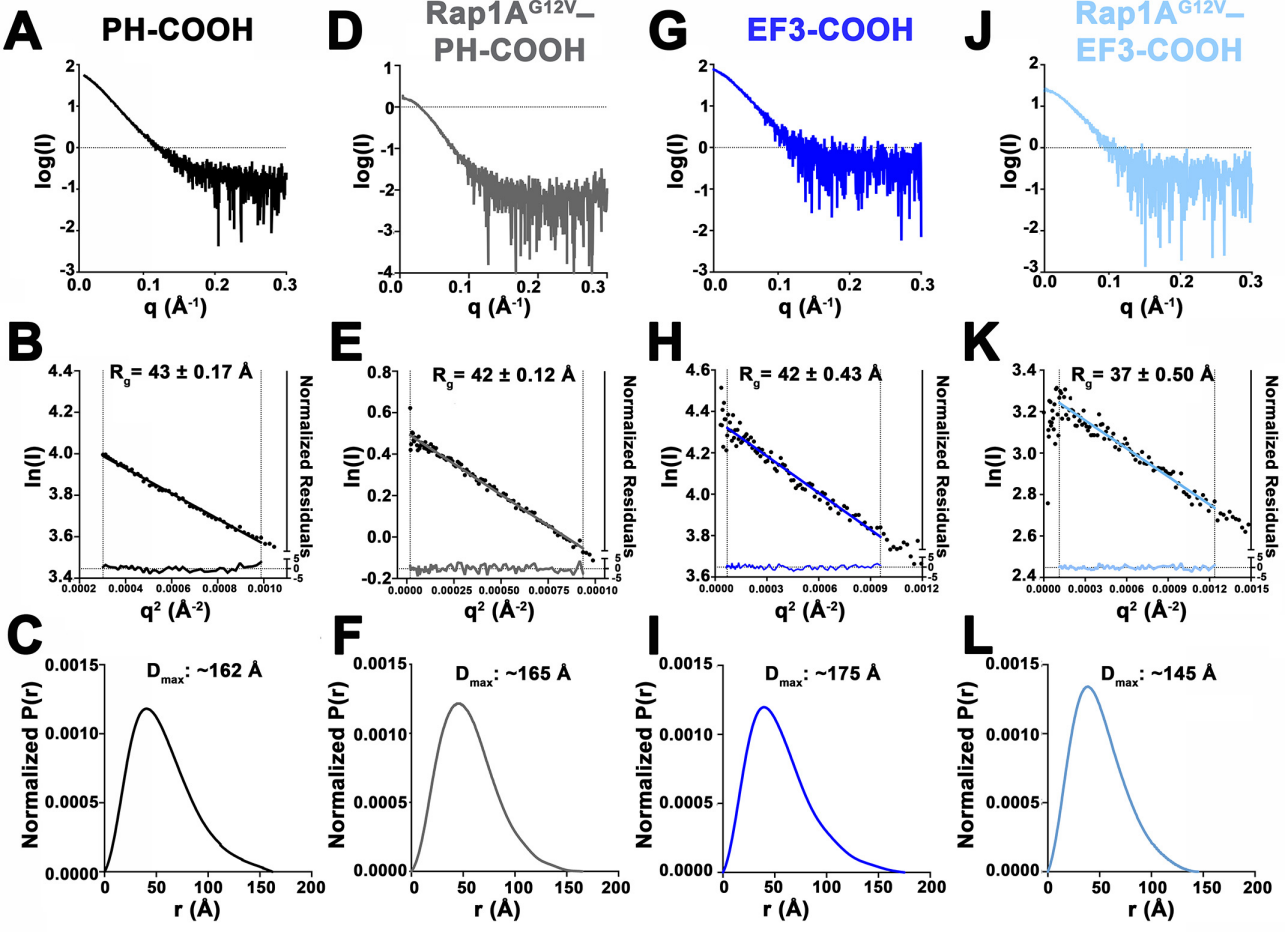
**Rap1A^G12V^ binding to PLCε PH-COOH or EF3-COOH stabilizes different conformational states.***A* and *B*, scattering profile for PLCε PH-COOH (*A*) and Guinier plot (*B*) demonstrate the variant is monomeric and monodisperse in solution. (*C*) Its pair–distance distribution function is consistent with a largely globular protein with some extended features. *D* and *E*, the scattering profile (*D*) and Guinier plot (*E*) for the Rap1A^G12V^–PH-COOH complex are also consistent with a monodisperse complex. (*F*) Its pair–distance distribution function shows a more compact structure upon the binding of Rap1A^G12V^. *G–I*, PLCε EF3-COOH is similar to PH-COOH in solution, as evidenced by its scattering profile (*G*), Guinier plot (*H*), and pair–distance distribution function (*I*). *J* and *K*, the Rap1A^G12V^–EF3-COOH complex does not have elevated lipase activity but is still monodisperse in solution as shown in (*J*) the scattering profile and (*K*) Guinier plot. (*L*) The shape of the pair–distance distribution function reveals the complex is more globular than EF3-COOH alone, and more compact, as evidenced by the smaller *D*_max_. The data for the PLCε PH-COOH variant are included for comparison (24).

**Figure 4:**
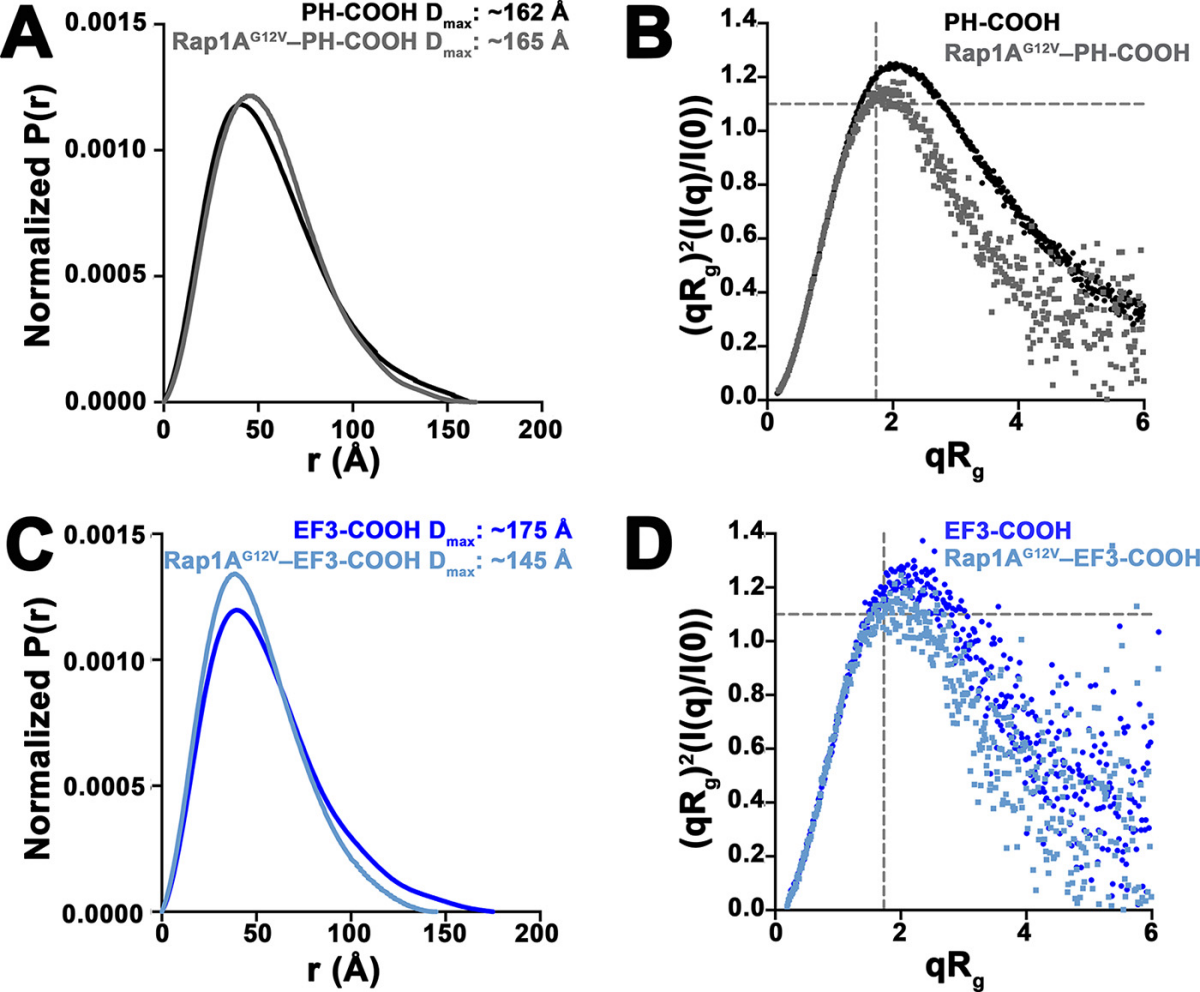
**Normalized pair–distance and dimensionless Kratky plots for PLCε variants alone and in complex with Rap1A^G12V^.***A*, the normalized *P*(*r*) functions for PH-COOH and Rap1A^G12V^–PH-COOH are similar, with *D*_max_ values of ∼162 and ∼165 Å, respectively. *B*, comparison of PH-COOH (*black circles*) and Rap1A^G12V^–PH-COOH (*gray squares*) shows that the complex is more compact and globular than PH-COOH alone, as evidenced by the more bell-shaped curve and convergence at lower qR_g_. *C*, the normalized *P*(*r*) functions for EF3-COOH and Rap1A^G12V^–EF3-COOH reveal that binding of Rap1A^G12V^ induces substantial conformational changes that lead to a more compact structure. This is further supported by the ∼30 Å decrease in *D*_max_ for the Rap1A^G12V^–EF3-COOH complex. *D*, comparison of EF3-COOH (*blue circles*) and Rap1A^G12V^–EF3-COOH (*light blue squares*). Rap1A^G12V^ binding induces conformational changes that result in a more compact and globular solution structure.

We first compared the SAXS solution structure of the Rap1A^G12V^–PH-COOH complex with that of PLCε PH-COOH. We previously showed this variant is a globular protein with extended features, likely because of the flexibly connected PH, EF1/2, and RA2 domains (Figure 3, Figure 4*A*, Table 2, Table S2, Table S3, and Fig. S2) (24, 31). The Rap1A^G12V^–PH-COOH sample was complicated and contained three minor components that partially overlapped the major peak corresponding to the complex in the SEC-SAXS elution profile (Fig. S2). Evolving factor analysis was used to deconvolute the data and identify the region corresponding to Rap1A^G12V^–PH-COOH (Fig. S3) (32, 33). The Rap1A^G12V^–PH-COOH complex had an *R*_g_ of 42.4 ± 0.12 Å (Fig. 3, *D* and *E*) and a *D*_max_ of ∼165 Å, with a largely globular structure with some extended features (Fig. 3). However, further analysis of the samples, shown in the dimensionless Kratky plot, revealed substantial conformational changes caused by Rap1A^G12V^ binding (Fig. 4*B*). In this plot, compact, globular proteins have bell-shaped curves that converge to the q*R*_g_ axis at low values, whereas elongated and more rigid structures exhibit curves that extend out to higher q*R*_g_, and highly flexible structures do not converge at all (34). The data for the Rap1A^G12V^–PH-COOH complex show that the overall structure is more compact and/or less flexible than PH-COOH alone, as evidenced by the curve being more bell-shaped and converging to 0 at lower values of q*R*_g_ (Fig. 4*B*).

**Table 2:** SAXS parameters of PLCε PH-COOH and EF3-COOH in complex with Rap1A^G12V^

	PH-COOH^a^	Rap1A^G12V^–PH-COOH	EF3-COOH	Rap1A^G12V^–EF3-COOH
**Guinier analysis**				
*I*(0) (Arb.)	64.92 ± 0.21	1.66 ± 0.003	78.10 ± 0.43	26.96 ± 0.50
*R*_g_ (Å)	42.7 ± 0.17	42.4 ± 0.12	41.9 ± 0.43	36.8 ± 0.50
*q* min (Å^−1^)	0.0174	0.0043	0.0084	0.0094
*q* range (Å^−1^)	0.0174–0.0314	0.0043–0.0306	0.0084–0.0309	0.0094–0.352
***P*(*r*) analysis**				
*I*(*0*) (Arb.)	65.96 ± 0.21	1.67 ± 0.03	79.43 ± 0.63	27.81 ± 0.21
*R*_g_ (Å)	44.79 ± 0.19	43.70 ± 0.16	44.48 ± 0.61	39.47 ± 0.43
*D*_max_ (Å)	162	165	175	145
Porod volume (Å^−3^)	191,000	237,000	179,000	178,000
q range (Å^−1^)	0.0174–0.308	0.0042–0.350	0.0087–0.387	0.0052–0.366


a The data for the PLCε PH-COOH variant was previously published, and is included for comparison (24).

We next compared the solution structures of PLCε EF3-COOH alone and in complex with Rap1A^G12V^. EF3-COOH was also monomeric and monodisperse in solution, with an *R*_g_ of 41.9 ± 0.43 Å and a *D*_max_ of ∼175 Å (Fig. 3, *G–I*, Table 2, Table S2, and Fig. S2). Its *P*(*r*) function is also consistent with the protein having a mostly globular structure with a modest degree of extendedness and/or flexibility, consistent with the flexibly connected RA2 domain (Figs. 3*I* and 4*C*). These results also confirm that deletion of the PH domain and EF1/2 in this variant does not perturb the EF3-RA1 structure in solution (Figure 3, Figure 4). Surprisingly, the solution structure of the Rap1A^G12V^–EF3-COOH complex differed substantially from EF3-COOH. The complex had an *R*_g_ of 36.8 ± 0.5 Å (Fig. 3, *J* and *K*, Table S2, and Fig. S2), and its *P*(*r*) function revealed a much more compact and globular structure, as shown by the more bell-shaped curve and most clearly in the ∼30 Å decrease in *D*_max_ to ∼145 Å (Figure 3, Figure 4 and Table 2). This is further highlighted in the dimensionless Kratky plot, which shows that Rap1A^G12V^ binding induces conformational changes that result in a more compact, stable structure (Fig. 4*D*). Because EF3-COOH is not activated by Rap1A^G12V^ (Fig. 1 and Table 1), this more condensed state may correspond to a nonproductive conformation of the complex in which only basal lipase activity is observed, or a state in which it is incompetent for activation, due to the loss of the PH domain and EF1/2.

## Discussion

The PLCε RA domains are highly similar in structure but have different functional roles in the enzyme (1, 6, 15, 16, 17, 18). The RA1 domain, together with the C2-RA1 linker, forms extensive contacts with EF3/4, the TIM barrel, and the C2 domain that are important for stability and activity (15). RA1 also interacts with mAKAP, a scaffolding protein at the perinuclear membrane, helping localize PLCε to internal membranes (18). The contribution of the RA2 domain to basal activity is unclear, because its deletion has been reported to either activate, inhibit, or have minimal impact (14, 15, 17). The RA2 domain is the primary binding site for activated Rap1A and Ras GTPases, because deletion of the domain or mutation of two highly conserved lysines (*R. norvegicus* PLCε Lys^2150^ and Lys^2152^; Fig. 2*A*) eliminates G protein–stimulated activation in cells (14, 17, 19). Interestingly, mutation of Lys^2150^ alone decreases basal activity ∼50% in cells (17, 19). NMR and biochemical studies have shown RA2 is flexibly connected to RA1 and does not stably associate with the PLCε core (14, 15). However, how GTPase binding to this domain is translated into increased lipase activity is poorly understood. Given that all known activators PLCε are lipidated, membrane localization is certainly one aspect of the activation mechanism. However, membrane association alone is insufficient to fully stimulate lipase activity. For example, a PLCε variant bearing a *CAAX* motif at its C terminus for constitutive plasma membrane localization had increased lipase activity, but was stimulated an additional ∼4-fold in the presence of activated Ras, suggesting that the activation mechanism mediated by small GTPases must also have an allosteric component (14).

In this work, we used a series of purified PLCε domain deletion variants and point mutants to investigate the allosteric component of the Rap1A-dependent activation mechanism. We have shown that constitutively active Rap1A binds to and increases the lipase activity of PLCε PH-COOH *in vitro* to a similar extent as full-length PLCε in cells (Fig. 1) (20, 21, 23). We also found the PH domain and EF1/2 are required for Rap1A-dependent activation (Fig. 1 and Table 1). These findings support a model in which the binding of Rap1A is a collaborative event involving multiple domains of PLCε.

We next sought to identify conserved residues on the RA2 domain, which is of known structure, that could be involved in mediating intramolecular interactions with the PLCε PH-RA1 core upon Rap1A binding. Guided by the previously determined crystal structure of the H-Ras–RA2 complex (Fig. 2*A*; PDB entry 2C5L (14)), we generated a model of the Rap1A–RA2 interaction and identified four conserved, solvent-exposed, hydrophobic resides on RA2 that are distant from the predicted Rap1A-binding site and mediated crystal contacts. Mutation of Tyr^2155^, Leu^2158^, Leu^2192^, or Phe^2198^ to alanine eliminated Rap1A^G12V^-dependent activation (Fig. 2 and Table 1). One explanation for these results is that the conserved, hydrophobic residues on the RA2 surface are needed to stabilize interactions between the Rap1A-bound RA2 domain and the PLCε core. Thus, the hydrophobic residues on RA2 may serve to communicate the fact that a GTPase is bound to the rest of the lipase.

**Figure 5:**
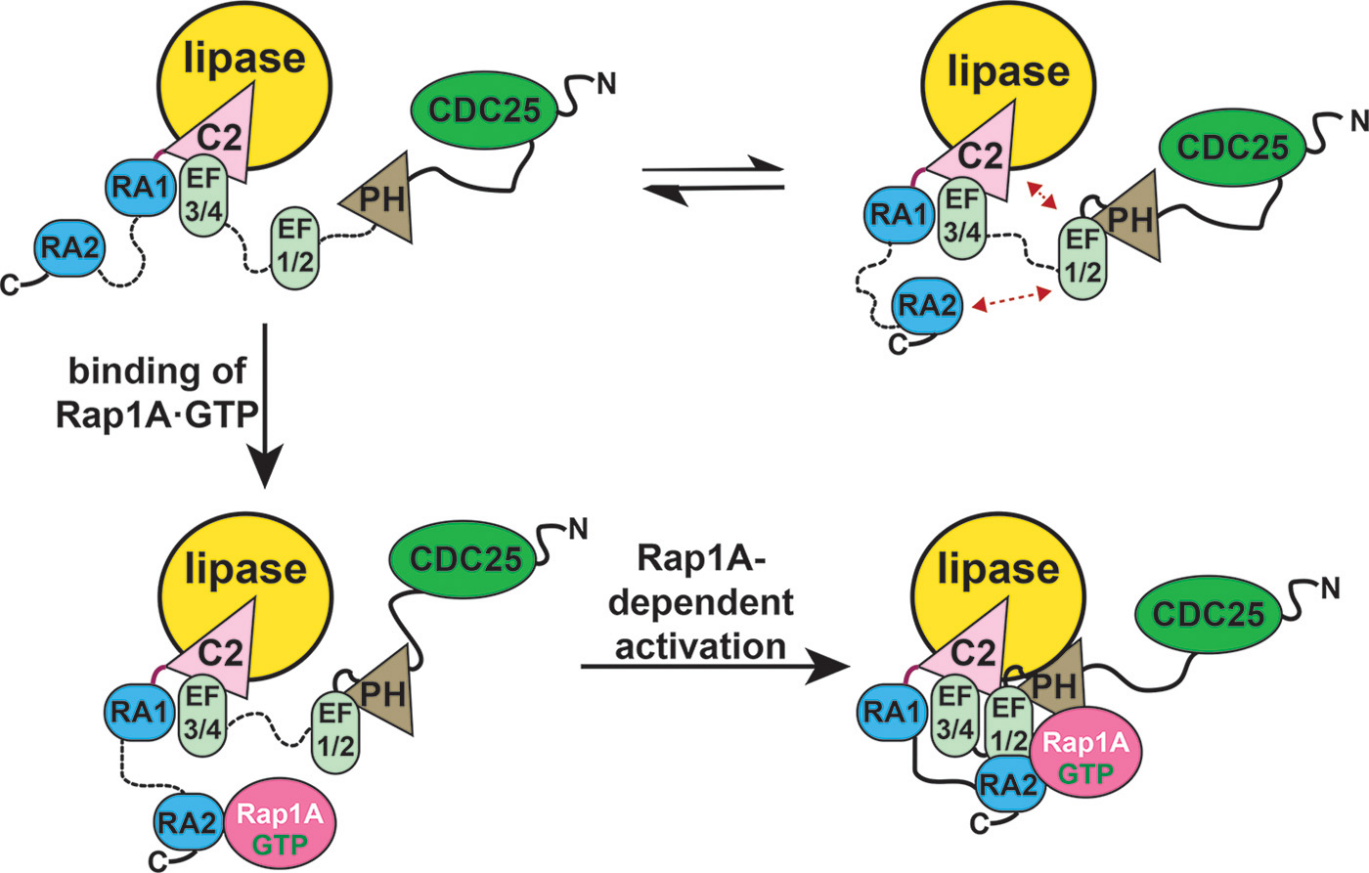
**A model for activation of PLCε by Rap1A.***Top panels*, PLCε exists in multiple conformational states in solution. The PH domain, EF1/2, and RA2 domains are flexibly connected to the rest of the enzyme, as indicated by the *dashed black lines*, and interact transiently with one another under basal conditions (*red arrows*) (24). *Bottom left panel*, activated Rap1A binds to its high-affinity binding site on the PLCε RA2 domain. However, this interaction is insufficient on its own to activate lipase activity. *Bottom right panel*, the Rap1A–RA2 complex also interacts with a site on the PLCε core, potentially formed by the PH domain and EF hands, resulting in Rap1A-dependent activation.

Our study demonstrates that the PLCε PH domain, EF1/2, and conserved hydrophobic residues on the RA2 surface are being for Rap1A-dependent activation. These domains are distant in the primary structure of PLCε (Fig. 1*A*) but may be in relatively close spatial proximity, based on the observed locations of the N and C termini in the recent structure of the PLCε EF3-RA1 fragment (15). To gain structural insights into how these elements could contribute to activation, we used SAXS to compare the solution structures of PLCε PH-COOH and EF3-COOH alone and in complex with Rap1A^G12V^. This comparison allows identification of large-scale conformational changes, changes in shape (globular *versus* extended), and differences in flexibility (Figure 4, Figure 5, Table 2, and Table S2). The PLCε variants alone had similar globular structures with some extended/flexible features, consistent with the presence of at least one flexibly connected domain. Binding of Rap1A^G12V^ to either variant induced conformational changes that resulted in more ordered and less flexible structures (Figs. 3, 4). However, these two Rap1A-bound complexes differ substantially from one another, because Rap1A^G12V^ binding to EF3-COOH also decreased the maximum diameter of the complex by ∼30 Å and stabilized a more globular structure (Figure 3, Figure 4 and Table 2). This smaller structure is likely due to the absence of the PH domain and EF1/2, which appear to be stabilized in an extended conformation in PH-COOH when Rap1A is bound, resulting in the similar maximum diameters and solution architectures of the PLCε variant alone and in complex with the GTPase (Figure 3, Figure 4 and Table 2). Stabilization may be achieved through intramolecular interactions between the hydrophobic surface of the RA2 domain and the PH domain and/or EF1/2.

Overall, our results provide the first direct structural evidence about the nature of the allosteric component of Rap1A-dependent (and potentially Ras-dependent) activation of PLCε and reveal that activation involves substantial conformational changes within the lipase. Whereas the PLCε RA2 domain is required for activation, this process also appears to be dependent on the PH domain, EF1/2, and hydrophobic surface residues on the RA2 domain. These observations are consistent with the following model (Fig. 5). Because the RA2 domain is flexibly tethered to RA1, it may transiently interact with rest the of PLCε core in solution. These interactions are insufficient to alter the thermal stability or increase basal activity. When Rap1A binds to the RA2 domain, the Rap1A-RA2 module could stably interact with other domains in PLCε. This most likely occurs through interactions between the hydrophobic residues on the RA2 surface and the PH domain and/or EF1/2. It is possible that the PH and RA2 domains are also in close proximity to one another, given the observed locations of the N and C termini in the PLCε EF3-RA1 crystal structure (15). Finally, the membrane itself contributes to Rap1A-dependent activation in several possible ways, such as through a membrane-induced allosteric event, and/or by stabilizing a unique and fully activated Rap1A–PLCε complex. Although the regions responsible for membrane association in PLCε have not yet been identified, the enzyme is able to at least transiently interact with the membrane, given its measurable basal activity (17). Rap1A is prenylated and thus, along with any other membrane binding element, helps orient the lipase active site at the membrane for maximum lipid hydrolysis. Future studies that provide higher resolution insights into the interactions between the RA2 domain and the PLCε core, alone and in complex with activated Rap1A, will be essential steps in elucidating a complete picture of this process and reveal new opportunities for therapeutic developments that target activation of PLCε by small GTPases.

## Experimental procedures

### Protein expression, purification, and mutagenesis of PLCε variants

cDNAs encoding N-terminally His-tagged *R. norvegicus* PLCε variants were subcloned into pFastBac HTA (PH-COOH, residues 837–2282; PH-C2, residues 832–1972; and EF3-COOH, residues 1284–2282). Site-directed mutagenesis in the PH-COOH background was performed using the QuikChange site-directed mutagenesis kit (Stratagene) or the Q5 site-directed mutagenesis kit (NEB). All subcloned PLCε variants contained an N-terminal His-tag and TEV cleavage site and were sequenced over the entire coding region. The proteins were expressed and purified as previously described (24), with some modifications for the PLCε EF3-COOH used in the SAXS experiments. Briefly, after elution from an Ni-NTA column, PLCε EF3-COOH was incubated with 5% (w/w) TEV protease to remove the N-terminal His-tag and dialyzed overnight against 1.5 liters of buffer containing 20 mm HEPES, pH 8.0, 50 mm NaCl, 2 mm DTT, 0.1 mm EDTA, and 0.1 mm EGTA at 4 °C. The dialysate was applied to Roche cOmplete Ni-NTA resin or a GE HisTrap, and the flow-through containing the TEV-cleaved EF3-COOH was collected and passed over the column two more times. The protein in the collected flow-through was then purified as previously described (24).

### Expression and purification of prenylated Rap1A^G12V^·GTP

cDNA encoding N-terminally His-tagged constitutively active *Homo sapiens* Rap1A (Rap1A^G12V^) was subcloned into pFastBac HTA. The protein were expressed in baculovirus-infected High5 cells. Cell pellets were resuspended in lysis buffer containing 20 mm HEPES, pH 8.0, 100 mm NaCl, 10 mm β-mercaptoethanol, 0.1 mm EDTA, 10 mm NaF, 20 mm AlCl_3_, 0.1 mm leupeptin and Lima Bean trypsin inhibitor, 0.1 mm phenylmethylsulfonyl fluoride, and 20 μm GTP and lysed via a dounce homogenizer on ice. The lysate was centrifuged for 1 h at 100,000 × *g*, and the pellet was resuspended in lysis buffer supplemented with 1% sodium cholate, resuspended via dounce homogenization on ice, and solubilized at 4 °C for 1 h. The sample was then centrifuged for 1 h at 100,000 × *g*, and the supernatant was diluted 2-fold with lysis buffer.

His-tagged Rap1A^G12V^ was loaded on a Ni-NTA column pre-equilibrated with lysis buffer and first washed with lysis buffer containing 10 mm imidazole and 0.2% cholate, followed by a second wash with lysis buffer supplemented with 10 mm imidazole and 10 mm CHAPS. The protein was eluted with lysis buffer containing 250 mm imidazole, pH 8.0, and 10 mm CHAPS. His-tagged Rap1A^G12V^ was then concentrated and applied to tandem Superdex S200 columns pre-equilibrated with G protein S200 buffer (20 mm HEPES, pH 8.0, 50 mm NaCl, 1 mm MgCl_2_, 2 mm DTT, 10 mm CHAPS, and 20 μm GTP). Fractions containing purified protein were identified by SDS-PAGE, pooled, concentrated, and flash-frozen in liquid nitrogen. Rap1A^G12V^ used for activation assays was purified in modified G protein S200 buffer containing 1 mm CHAPS.

For SAXS experiments, His-tagged Rap1A^G12V^ was incubated with 5% (w/w) TEV protease and dialyzed overnight in 1.5 liters of dialysis buffer containing 20 mm HEPES, pH 8.0, 50 mm NaCl, 1 mm MgCl_2_, 10 mm β-mercaptoethanol, 1 mm CHAPS, and 20 μm GTP at 4 °C. The dialysate was applied to a Ni-NTA column, and the flow-through containing cleaved Rap1A^G12V^ was collected and passed over the column two more times. The flow-through was collected, concentrated to 1 ml, applied to tandem Superdex S200 columns, and purified as described above.

### Differential scanning fluorimetry

Melting temperatures (*T*_m_) of PLCε variants were determined as previously described (24, 30). A final concentration of 0.5 mg/ml was used for each PLCε variant. At least three independent experiments were performed in duplicate.

### PLCε activity assays

All activity assays were carried out using [^3^H]PIP_2_ as the substrate. Basal activity of PLCε variants was measured as previously described (24). Briefly, 200 μm phosphatidylethanolamine, 50 μm PIP_2_, and ∼4,000 cpm [^3^H]-PIP_2_ were mixed, dried under nitrogen, and resuspended by sonication in buffer containing 50 mm HEPES, pH 7, 80 mm KCl, 2 mm EGTA, and 1 mm DTT. Enzyme activity was measured at 30 °C in 50 mm HEPES, pH 7, 80 mm KCl, 15 mm NaCl, 0.83 mm MgCl_2_, 3 mm DTT, 1 mg/ml BSA, 2.5 mm EGTA, 0.2 mm EDTA, and ∼500 nm free Ca^2+^. PLCε PH-COOH and EF3-COOH were assayed at a final concentration of 0.075 ng/μl and PH-C2 at 0.1–1 ng/μl (24). The PH-COOH K2150A, K2152A, Y2155A, L2158A, L2192A, and F2198A mutants were assayed at a final concentration of 0.5 ng/μl. Control reactions contained everything except free Ca^2+^. The reactions were quenched by the addition of 200 μl of 10 mg/ml BSA and 10% (w/v) ice-cold TCA and centrifuged. Free [^3^H]IP_3_ in the supernatant was quantified by scintillation counting. All assays were performed at least three times in duplicate.

Rap1A^G12V^-dependent increases in PLCε lipase activity were measured using the same approach with some modifications. The liposomes were first incubated with increasing concentrations of Rap1A^G12V^·GTP in 50 mm HEPES, pH 7.0, 3 mm EGTA, 1 mm EDTA, 100 mm NaCl, 5 mm MgCl_2_, 3 mm DTT, and 390 μm CHAPS at 30 °C for 30 min. The reaction was initiated by addition of the PLCε variant, incubated at 30 °C for 8 min, and processed as described above. All activation assays were performed in duplicate with protein from at least two independent purifications.

### Formation and isolation of the Rap1A^G12V^–PLCε variant complexes

A 1:3 or 1:5 molar ratio of PLCε PH-COOH or EF3-COOH to Rap1A^G12V^, supplemented with 0.5 mm CaCl_2_, was incubated on ice for 30 min before being applied to a Superdex S200 column pre-equilibrated with complex S200 buffer (20 mm HEPES, pH 8, 50 mm NaCl, 2 mm DTT, 0.1 mm EDTA, 0.1 mm EGTA, 1 mm MgCl_2_, 0.5 mm CaCl_2_, and 40 μm GTP). Fractions containing the purified complex were identified by SDS-PAGE, pooled, and concentrated for use in SAXS experiments.

### SAXS data collection and analysis

PLCε PH-COOH was previously characterized by SAXS (24). PLCε EF3-COOH, the Rap1A^G12V^–PLCε PH-COOH complex, and the Rap1A^G12V^–PLCε EF3-COOH complex were diluted to final concentrations of 2–3 mg/ml in S200 buffer (EF3-COOH) or complex S200 buffer and centrifuged at 16,000 × *g* for 5 min at 4 °C prior to data collection. SEC-SAXS was performed at the BioCAT Beamline at Sector 18 of the Advanced Photon Source (Table S3).

Protein samples were eluted from a Superdex 200 Increase 10/300 GL column using an ÄKTA Pure FPLC (GE Healthcare) at a flow rate of 0.7 ml/min. The eluate passed through a UV monitor followed by a SAXS flow cell consisting of a quartz capillary. The data were collected in two different setups at the beamline. The PH-COOH, EF3-COOH, and EF3-COOH complex data were collected in a 1.5-mm–inner diameter quartz capillary with 10 μm walls. The PH-COOH complex data were collected in a 1.0-mm–inner diameter quartz capillary with 50 μm walls using the co-flow sample geometry (35) to prevent radiation damage. Scattering intensity was recorded using Pilatus3 X 1M detector (Dectris) placed ∼3.7 m from the sample using 12 KeV X-rays (1.033 Å wavelength) and a beam size of 160 × 75 μm, giving an accessible *q* range of ∼0.004–0.36 Å^−1^. Data were collected every 2 s with 0.5-s exposure times. The data in regions flanking the elution peak were averaged to create buffer blanks, which were subsequently subtracted from exposures selected from the elution peak to create the final scattering profiles (Fig. S2). BioXTAS RAW 1.4.0(33) was used for data processing and analysis. For the Rap1A^G12V^–PLCε PH-COOH complex, the four components present in the sample were deconvoluted using evolving factor analysis (Fig. S3) (32) as implemented in BioXTAS RAW (33). The radius of gyration (*R*_g_) of individual frames were plotted with the scattering chromatograms, which show the integrated intensity of individual exposures as a function of frame number, and used to help determine appropriate sample ranges for subtraction. PRIMUS (36) was used to calculate the *R*_g_, *I*_(0)_, and *D*_max_. GNOM (37) was used within PRIMUS to generate the pair–distance distribution (*P*(*r*)) functions via an indirect Fourier transform method.

Graphical plots were generated from buffer-subtracted averaged data (scattering profile and Guinier plots) (38) or indirect Fourier transform data (P(r) plots) and plotted using GraphPad Prism v.8.0.1. SAXS data are presented in accordance with the publication guidelines for small-angle scattering data (39).

### Statistical methods

GraphPad Prism v.8.0.1 was used to generate all plots. One-way analysis of variance was performed with Prism v.8.0.1 followed by Dunnett's post hoc multiple comparisons *versus* PLCε PH-COOH, as noted in the figure captions. All *error bars* represent standard deviation.

## Data availability

Data not provided within the article is available upon reasonable request to A. M. Lyon at lyonam@purdue.edu
