# Supplementary material for: Functional and structural characterization of allosteric activation of phospholipase Cε by Rap1A
Source: J Biol Chem. 2021 Jan 13;295(49):16562–71. doi: 10.1074/jbc.RA120.015685 (PMC7864056; doi:10.1074/jbc.RA120.015685)
Supplement: Supplementary file 1 [file mmc1.pdf]

SUPPORTING INFORMATION

Functional and structural characterization of allosteric activation of Phospholipase Cε by Rap1A

Monita Sieng[†#], Arielle F. Selvia[†], Elisabeth E. Garland-Kuntz[†], Jesse B. Hopkins[‡], Isaac J. Fisher[†], Andrea T. Marti[†], and Angeline M. Lyon[†§]*

[†]Department of Chemistry and [§]Department of Biological Sciences, Purdue University, West Lafayette, Indiana 47907, United States [1]and the [‡]Biophysics Collaborative Access Team, Illinois Institute of Technology, Sector 18ID, Advanced Photon Source, Argonne National Laboratory, Lemont, IL 60439

Present Addresses: [#]Sequlite Genomics, 6773 Sierra Ct, Dublin, CA 94568

*Corresponding author: Angeline M. Lyon

Email: lyonam@purdue.edu

Supporting Table 1. Minimum and Maximum Specific Activities under Rap1A^{G12V} Activation Conditions

PLCε variants	Minimum activity ± SD (nmol IP_3/min/nmol PLCε variant)	Maximum Rap1A^{G12V}-stimulated activity (nmol IP_3/min/nmol PLCε variant) (n)
PH-COOH	630 ± 100	1,900 ± 300 (4)
PH-C2	70 ± 30	24 ± 9 (3)
EF3-COOH	335 ± 80	200 ± 15 (2)
PH-COOH K2150A	420 ± 70	540 ± 200 (2)
PH-COOH K2152A	590 ± 200	700 ± 100 (3)
PH-COOH Y2155A	520 ± 50	640 ± 30 (3)
PH-COOH L2158A	710 ± 200	780 ± 70 (3)
PH-COOH L2192A	430 ± 200	960 ± 30 (3)
PH-COOH F2198A	380 ± 100	630 ± 300 (3)

These specific activities are determined at a single time point.

Supporting Table 2. SAXS Molecular Weight Estimations for PLCε PH-COOH and EF3-COOH alone and in complex with Rap1A^{G12V}

	PH-COOH[1]	Rap1A^{G12V}–PH-COOH	EF3-COOH	Rap1A^{G12V}–EF3-COOH
P(r) analysis				
Porod Volume MW (kDa), (ratio to expected)	130 (0.76)	200 (1.0)	150 (1.3)	120 (0.88)
Volume of Correlation MW (kDa), (ratio to expected)	120 (0.71)	160 (0.82)	120 (1.1)	100 (0.73)
Calculated MW (kDa)	165.5	189.8	112.8	134.2

[1] The data for the PLCε PH-COOH variant was previously published[22], and is included here for comparison.

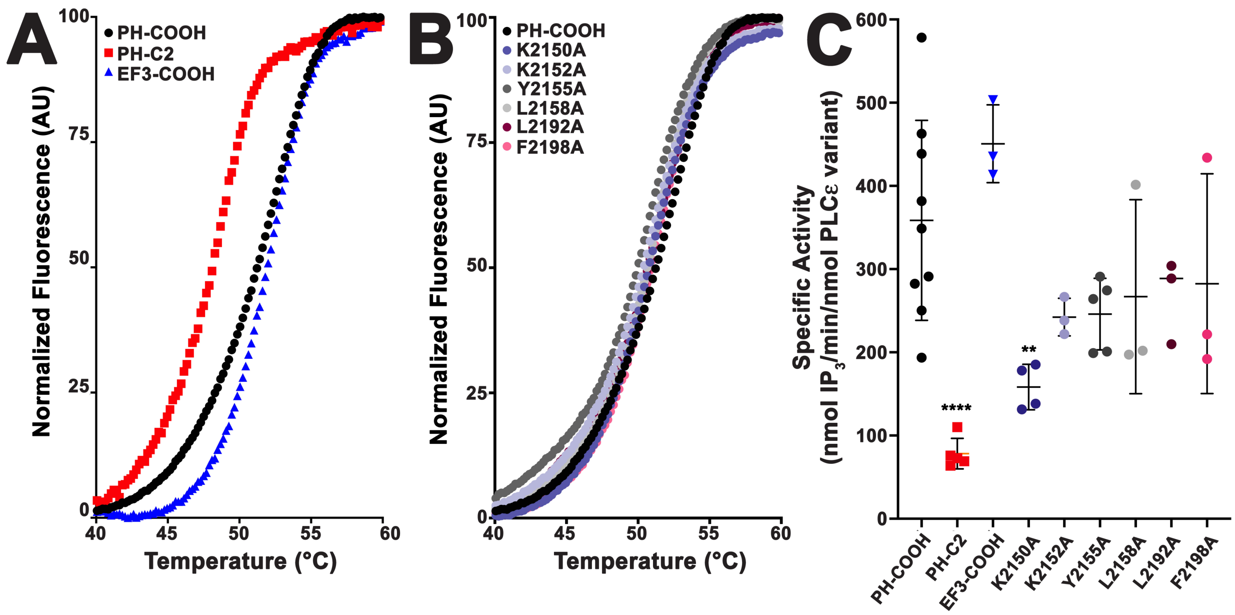

Supporting Figure 1. Characterization of the PLCε domain deletion variants and RA2 point mutants. (A) Representative thermal denaturation curves of the PLCε domain deletion variants used in this study. PH-COOH is shown in black circles (23), PH-C2 in red squares (23), and EF3-COOH in blue triangles. (B) Representative thermal denaturation curves the PH-COOH RA2 point mutants. PH-COOH is shown as in (A), K2150A in navy blue circles, K2152 in periwinkle circles, Y2155A in gray circles, L2158A in light gray circles, L2192A in maroon circles, and F2198A in pink circles. (C) Scatter plot of the basal specific activities of the PLCε variants. All assays were performed at least three times in duplicate, and error bars represent SD. Significance was determined using a one-way ANOVA followed by Dunnett's multiple comparisons test vs. PLCe PH-COOH. **** $p \leq 0.0002$, ** $p \leq 0.0028$.

Supporting Table 3. SAXS Data Collection and Analysis Parameters

(a) SAXS data collection parameters	
Instrument	BioCAT facility at the Advanced Photon Source beamline 18ID with Pilatus3 X 1M (Dectris) detector
Wavelength (Å)	1.033
Beam size (mm^2)	160 (h) x 75 (v)
Camera length (m)	3.7
q-measurement range ($Å^{-1}$)	0.004-0.4
Absolute scaling method	N/A or Glassy Carbon, NIST SRM 3600
Basis for normalization to constant counts	To incident intensity, by ion chamber counter
Method for monitoring radiation damage	Automated frame-by-frame comparison of relevant regions using CORMAP[35] implemented in BioXTAS RAW
Exposure time, number of exposures	0.5 s exposure time with 2 s exposure period (0.5 s on, 1.5 s off) of entire SEC elution
Sample configuration	SEC-SAXS. Separation by size using ÄKTA Pure with a Superdex 200 Increase 10/300 GL column. Some SAXS data measured in a 1.5 mm ID quartz capillary while others used a sheath-flow cell[24] with effective path length 0.542 mm.
Sample temperature (˚C)	20
(b) Software employed for SAXS data reduction, analysis and interpretation	
SAXS data reduction	Radial averaging; BioXTAS RAW 1.4.0[25] and ATSAS[29] used for frame comparison, averaging, and subtraction
Basic analysis: Guinier, M.W., P(r)	BioXTAS RAW 1.4.0[25] used for Guinier fit and molecular weight; GNOM[36] used for P(r) function

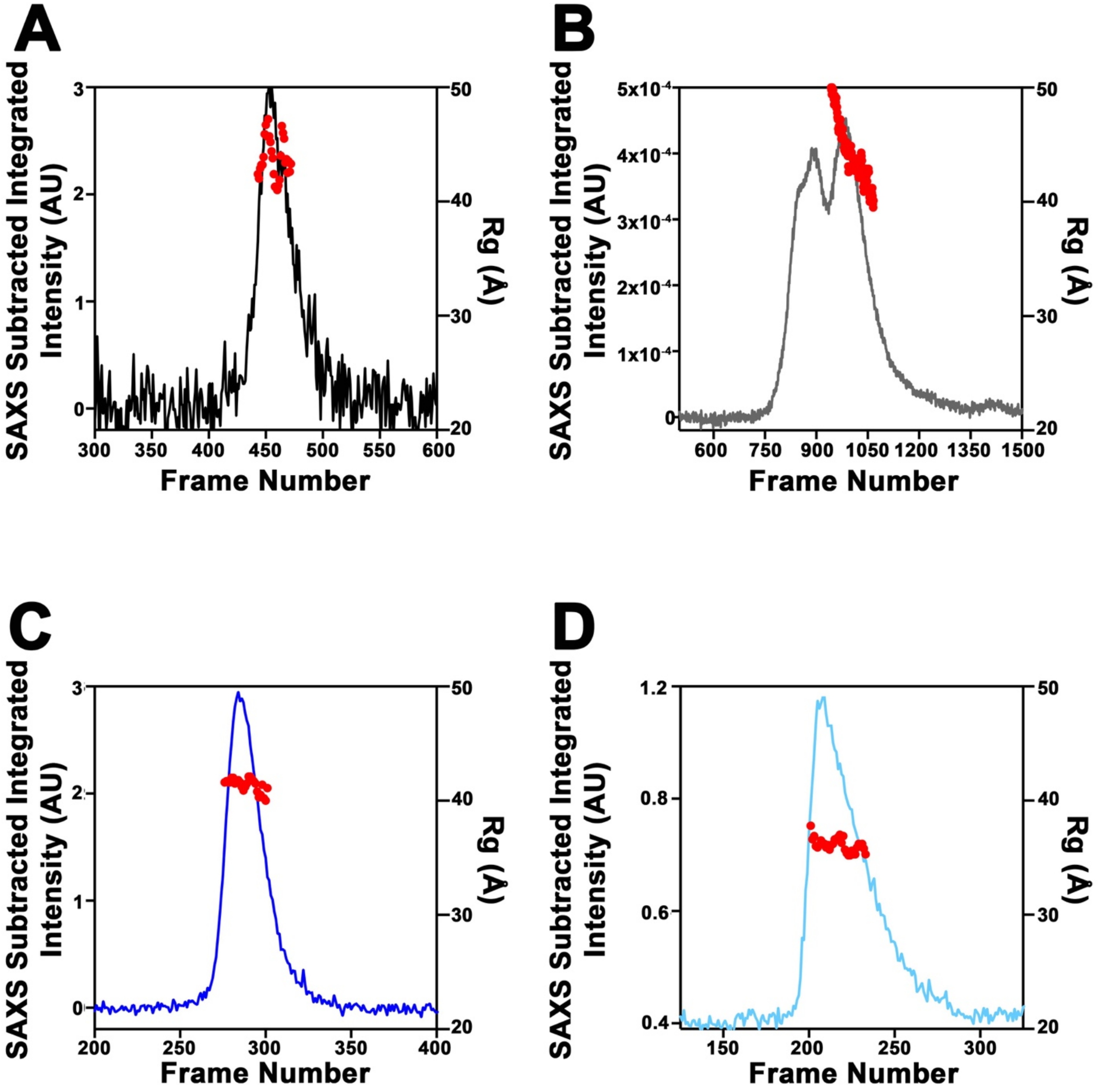

Supporting Figure 2. Size exclusion chromatography (SEC-SAXS) scattering chromatograms for PLCε variants alone and in complex with Rap1A^{G12V}. Each chromatogram shows the SAXS subtracted integrated intensity and R_g as a function of exposure (frame) number for (A) PLCε PH-COOH (23), (B) Rap1A^{G12V}–PH-COOH, (C) PLCε EF3-COOH, and (D) Rap1A^{G12V}–EF3-COOH. Red circles correspond to R_g.

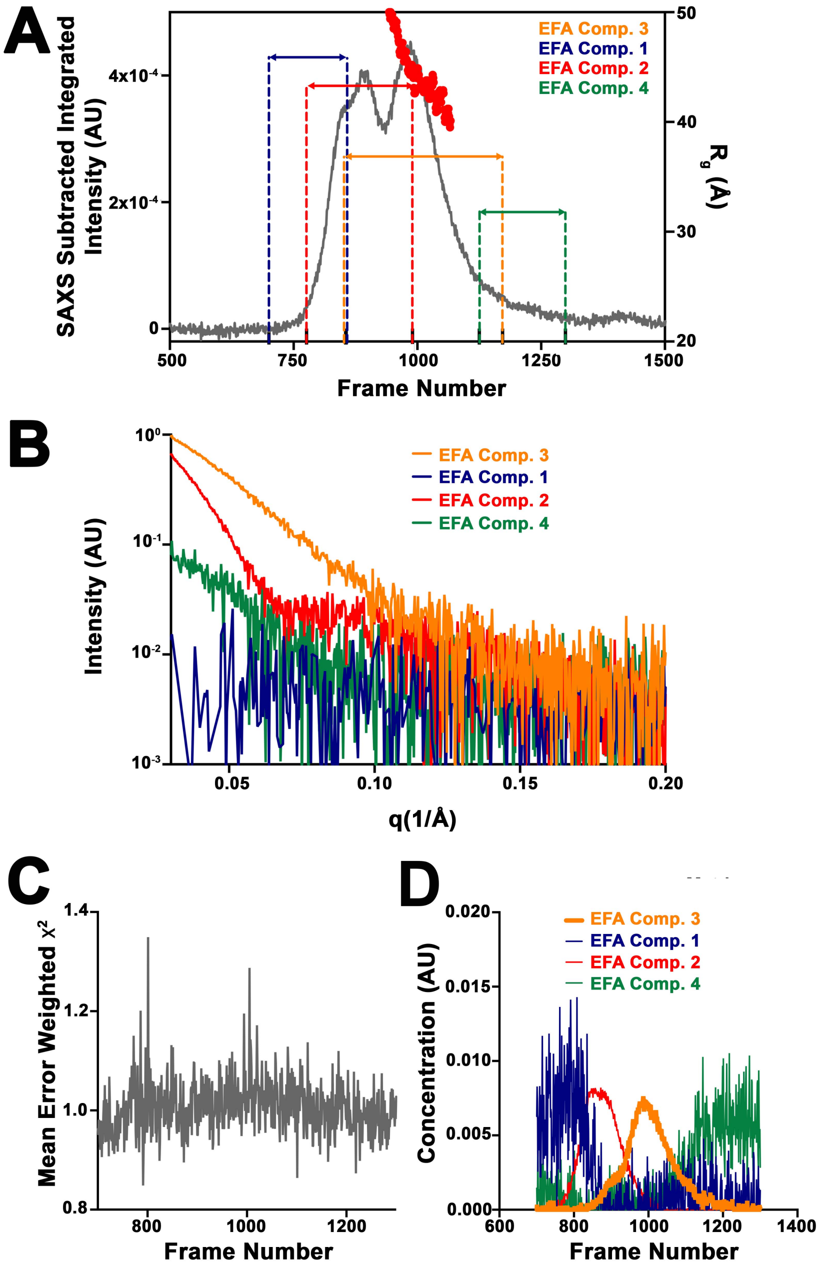

Supporting Figure 3. Deconvolution of the Rap1A^{G12V}–PH-COOH elution peak using evolving factor analysis (EFA). (A) Size exclusion chromatogram (SEC-SAXS) scattering chromatogram showing the SAXS subtracted integrated intensities and R_g as a function of frame number. Four components were identified in the Rap1A^{G12V}–PH-COOH sample (EFA Comp. 1-4), and their corresponding ranges are shown in dashed lines. EFA Comp. 3 (orange) corresponds to Rap1A^{G12V}–PH-COOH. (B) Overlaid SAXS scattering curves for each component identified by EFA. (C) Mean error weighted χ^2 of the EFA deconvolution. Values near 1 show a good fit of the deconvolution results to the original data. (D) Area normalized concentration profiles extracted from EFA for each component, colors correspond to region colors in A. This shows clearly where in the elution each component comes from, and that each component represents a relatively pure species.
